# Design and Heat Transfer Analysis of Graphene-Based Electric Heating Solid Wood Composite Energy Storage Flooring

**DOI:** 10.3390/ma18030698

**Published:** 2025-02-05

**Authors:** Bo Guan, Wen Qu, Xinchi Tian, Zihao Zhang, Guoyu Sun, Siman Zhou, Xiaoyu Feng, Chengwen Sun, Chunmei Yang

**Affiliations:** College of Mechanical and Electrical Engineering, Northeast Forestry University, Harbin 150040, China

**Keywords:** graphene electric heating material, composite flooring, heat transfer model, energy storage material

## Abstract

Due to severe global energy issues and the widespread demand for high-quality winter heating, this study designed a new type of graphene-based electrically heated solid wood composite floor. This flooring maintains the convenience of a traditional floor installation while providing users with a more comfortable living experience. Additionally, the low-temperature heating and temperature regulation system further reduces energy consumption, offering a new perspective for green home living. This paper introduces the overall structure and temperature control system of the graphene-heated solid wood composite flooring. Based on the above reasons, the working mechanism and heat transfer process of the graphene-heated flooring were analyzed, and a mathematical model was established. Furthermore, simulations of flooring with different thicknesses were conducted to determine temperature rise curves and corresponding times. Finally, a comparative experimental verification was conducted on the thermodynamic performance of the solid wood composite graphene flooring. The results showed that in the case of a floor with an 18 mm thickness, the time for the surface layer of the floor to reach 22 °C is 27 min; the time to reach 26 °C is 56 min; and that the time to reach 28 °C is 109 min. The time required to return to 22 °C after the power has been switched off is 25 min. The results also showed that one hour after the power was turned off, the surface temperature of the floor was still above 20 °C. The study shows that the graphene-heated flooring can be used to achieve high-quality heating.

## 1. Introduction

In regions with relatively severe cold weather in global, temperature and the quality of heating during winter is a widespread concern among people. While ensuring adequate temperature which people have started to set certain requirements for comfort [1,2]. Electrically heated floors that use materials to create greater comfort, that have a reasonable and efficient internal structure, and that allow for the adoption of more energy-saving heating methods have become important directions for the flooring industry. Exploring new heating methods and achieving efficient energy utilization are also important tasks for China to cope with the energy crisis and fulfill its commitments to the “Copenhagen Climate Agreement” and “carbon peak and carbon neutrality” [3,4,5].

In a study on efficient heating and low-temperature flooring, Zhou Yucheng proposed various structures to enhance the thermal comfort and heat transfer efficiency of electrically heated floor heating while reducing safety hazards. These included, as follows: a composite structure of solid wood flooring and aluminum alloy core-board for electric heating [6]; a solar energy storage flooring [7] and a polyurethane flooring structure [8]. Zhang et al. [9,10,11] revealed the heat transfer mechanism of low-temperature hot water floor radiant heating through experiments. The results indicated that the longitudinal temperature difference was small, resulting in minimal indoor temperature fluctuations and a uniform distribution. Compared to other heating methods, such as air conditioning and radiators, low-temperature hot water floor radiant heating offers significant advantages in terms of indoor thermal comfort. Luo et al. [12] employed a discrete phase model based on the Euler–Lagrange method to establish a heat transfer model coupling capillary floor radiant heating systems with indoor particle diffusion. They analyzed the temperature of the floor surface under different floor finishes and the concentration distribution characteristics of indoor rooms of different sizes and optimized the system. Their findings are of great significance for improvements in floor structures. Cui et al. [13] conducted experiments and research on the flow and dispersion characteristics caused by the combination of indoor floor radiant heating and natural ventilation. In this study, a reduced-scale numerical model validated by wind tunnel experiments was used to investigate the impact of combining floor radiant heating with natural ventilation on airflow, heat transfer, and pollutant diffusion in isolated buildings. Hervás-Blasco et al. [14] experimentally studied the thermal performance of a low-temperature hot water floor radiant heating system using a heat pump as the heat source. It was shown that the carbon dioxide emissions of this system are only 80% of those from traditional gas boilers used as heat sources, and it provides good comfort and low carbon emissions during use and operation. Cho et al. [15] studied a pre-control system for floor radiant heating systems based on experiments and numerical simulations. The results showed that after applying this system, the entire heating system achieved energy savings of about 10% and the upgraded system had greater economic efficiency. Balandin et al. [16] established a low-temperature hot water floor radiant heating system model through a software simulation, summarizing parameters that enhance thermal comfort and energy efficiency, demonstrating the advantages of floor heating in practical applications and providing reliable quantitative experimental data and evidence. Graphene is composed of a single layer of atoms and possesses a unique crystal structure. Its strong C-C bonding and complete in-plane crystal structure endow it with exceptionally high in-plane thermal conductivity [17], surpassing that of even silver, which has the best thermal conductivity among metals. Additionally, graphene exhibits an ultra-high surface emissivity, allowing for further enhancement of its heat dissipation performance through radiative cooling. It is regarded as an ideal material for novel and efficient heat transfer applications. Table 1 compares the thermal conductivity of graphene and metallic materials.

The aforementioned research indicates that solid wood composite graphene electric heating flooring represents an emerging means of heating that utilizes a more efficient heat conduction method. Coupled with energy-saving and intelligent feedback control systems, it is a viable primary heat source for indoor heating [5]. The graphene electric heating solid wood composite flooring designed in this study has undergone improvements in material selection and internal structural design compared to previous models, thereby enhancing the heat transfer efficiency of the electric heating floor to a certain extent. The heating principle and effect are quite similar to the “warming the feet while cooling the head” principle advocated in Chinese health preservation practices [18]. This study proposes a novel structure for graphene electric heating solid wood composite flooring and conducts a heat transfer analysis on the flooring to obtain temperature change data. The rationale and scientific design of the floor were theoretically proven and the impact of the innovative materials and structures on thermal efficiency were explored.

## 2. Overall Design of Composite Graphene Heating Floor

Engineered wood flooring is a type of tongue-and-groove flooring made with solid wood boards or veneers as the surface layer, solid wood as the core layer, and veneers as the bottom layer. The name of the flooring species is usually determined by the surface layer species. It differs from the so-called “composite flooring” on the market, as engineered wood flooring is made by laminating boards of different tree species in a staggered manner, and thus engineered flooring overcomes the disadvantage of the unidirectional homogeneity of solid wood flooring. It has a low rate of shrinkage and expansion, good dimensional stability, and retains the natural wood grain and comfortable foot feel of solid wood flooring. This floor combines the stability of laminate flooring with the beauty of solid wood flooring and has considerable environmental advantages.

### 2.1. Structural Design of Composite Graphene Heating Floor

Referring to Standard GB/T 18103-2022 [19], the graphene–electric heating solid wood composite floor studied in this study adopts a three-layer structure, The panel layer is double-sided sanded to a thickness of ≥2 mm, using veneer crafted from cherry wood, laid in the grain direction; the substrate layer is made of ordinary pine, sand-finished to a thickness of 8 mm, laid in the cross-grain direction. The bottom layer is a low-grade veneer, using veneer made from rotary-cut pine, double-sided sanded to a thickness of 2 mm. A 2 mm-thick thermal insulation asbestos mesh is selected and a groove with a depth of 2 mm is processed on the lower surface of the substrate layer. The asbestos mesh is inserted into the groove and the lower surface of the substrate is closely adhered to the upper surface of the bottom plate using a urea–formaldehyde resin adhesive. Graphene layers are added to the surface layer and substrate layer for heat conduction. The bottom of the panel layer is opened with a 2 mm-deep ribbed groove. Based on the heat transfer principle of fins, the graphene heat dissipation plate is processed with protruding ribs on the upper surface to increase the heat transfer area. The ribbed part of the graphene layer is embedded in the groove, and the upper end surface of the graphene layer is evenly coated with 0.5 mm thick urea–formaldehyde resin adhesive. The adhesive here serves both as a bonding agent and as an insulating layer for graphene. The lower surface of the graphene layer is adhered to the upper surface of the substrate layer using the urea–formaldehyde resin adhesive. The graphene layer consists of an upper insulating layer that a ribbed graphene heat dissipation layer, an electric heating film, and a lower graphene heat dissipation layer. The electric heating film is made of 0.250PET + 0.050EVA + 0.100PET, with an actual thickness of approximately 0.5 mm. The lower end surface of the electric heating film is adhered to the upper end surface of the lower graphene heat dissipation layer, and no groove is processed on the lower graphene heat dissipation layer. The lower end surface of the lower graphene heat dissipation layer is also coated with 0.5 mm thick urea–formaldehyde resin adhesive, serving as both a bonding agent and an insulating layer. The overall graphene layer is partially installed in the groove of the substrate layer and partially adhered to the groove on the lower end surface of the surface layer, with the surface layer and substrate layer glued together using urea–formaldehyde resin. The overall specifications are 910 mm × 127 mm × 18 mm.

**Figure 1:**
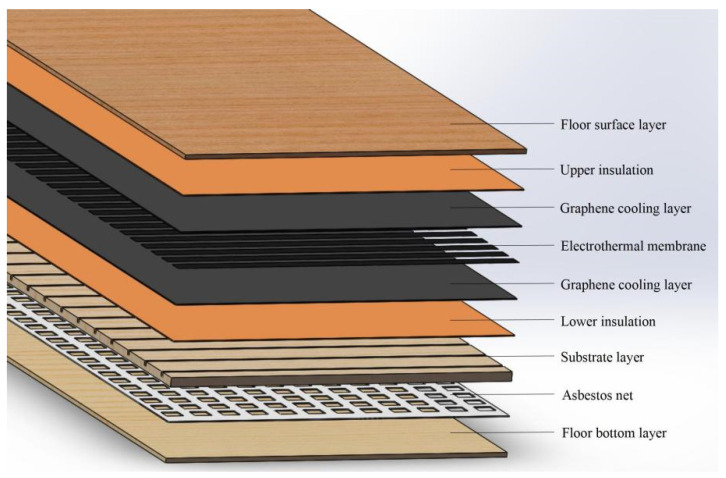
Hierarchical structure of graphene composite floor.

The layered structure of the graphene-heated solid wood composite flooring is shown in Figure 1.

After the graphene-based electric heating composite floor is powered on, heat is transferred to the graphene heat dissipation panel through the electric heating film. According to the second law of thermodynamics, heat flows in both upward and downward directions. The heat transferred upward passes through the graphene ribs to the surface panel and is then conducted into the indoor air through thermal convection and radiation [20]. A portion of the heat transferred downward is stored in the substrate layer after being insulated by the asbestos mesh, achieving an energy storage effect. Metal carbon crystal–graphene is a lightweight material with excellent heat dissipation properties, good oxidation resistance, and a low cost. It is generally used as the preferred material for thermal conduction and heat dissipation [21,22,23]. Therefore, the composite graphene-based electric heating floor discussed in this study uses carbon crystal–graphene film as the thermal conduction material. Additionally, asbestos insulation is selected as the thermal insulation layer for the composite graphene-based electric heating floor because asbestos fibers have a thermal conductivity of 0.104–0.260 W/(m·k) [24], good thermal insulation properties, high fire resistance, high tensile strength, chemical and thermal corrosion resistance, and good electrical insulation and thermal insulation properties. These characteristics perfectly meet the performance requirements of the composite graphene-based electric heating floor discussed in this study [25].

**Figure 2:**
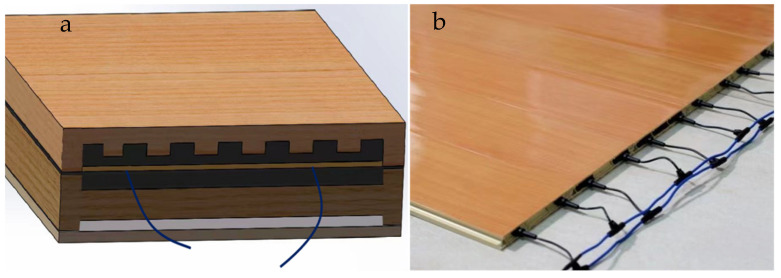
Overall structure of graphene electric heating floor: (**a**) three-dimensional structure of electric heating floor; and (**b**) electric heating floor lead.

This study uses 3D design software Solidworks 2016 to create a model of graphene–electric heated flooring, shown in Figure 2. The surface veneer is mortised with the graphene radiator panel, while the base material layer is glued to the floor. The lower end surface of the base material is slotted to embed a heat-insulating asbestos mesh.

As shown in Figure 2, the model of the established solid wood composite graphene electric heating floor is composed of, from top to bottom the following layers: the solid wood floor surface layer; the upper insulation layer; the graphene heat dissipation plate; the asbestos insulation layer; and the solid wood floor bottom layer. The purpose of grooving the top and bottom of the carbon crystal–graphene heat dissipation plate is to expand the area of the heat transfer surface. According to Fourier’s law of heat conduction, the heat transfer quantity Q is proportional to the heat transfer area of the carbon crystal–graphene heat dissipation plate. Therefore, increasing the heat transfer area can enhance the amount of heat transfer. This study designs the carbon crystal–graphene heat dissipation plate based on the theory of ribbed heat transfer, where the protruding surface of carbon crystal–graphene becomes a rib [26] that can effectively achieve the purpose of increasing the amount of heat transfer.

### 2.2. Design of Temperature Control System for Graphene Electric Heating Floor

**Figure 3:**
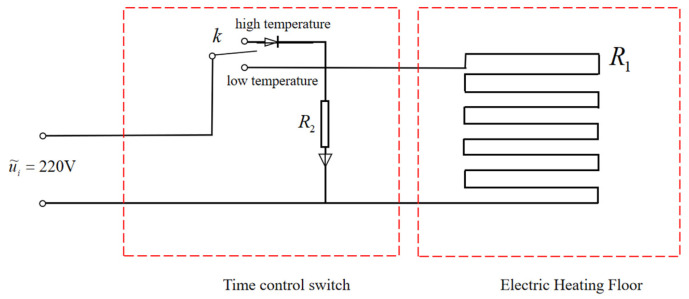
Graphene electric heating floor control system.

This study aims to design an automatic temperature regulation and control system for graphene electric heating flooring, enabling the floor to self-regulate the heating temperature during operation. The designed temperature control system primarily comprises, as follows: a thermal energy generation system; a thermal energy conversion system; a thermal energy transfer system; a heat dissipation system; and an automated control system for maintaining the indoor temperature through a timer. The implementation process of the control system is as follows. After being connected to the power supply, the electric heating film generates heat and adjusts the required heating time through the timer. Subsequently, the heat is transferred to the surface of the solid wood floor via the carbon crystal–graphene film. The electric heating control system for the graphene electric heating floor is shown in Figure 3.

**Figure 4:**
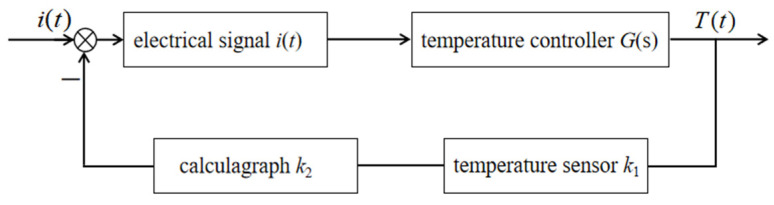
Transfer function of control system.

Using a timer to detect the time and convert it into a voltage signal, this signal is applied to the input of an amplifier along with a voltage signal corresponding to a preset time. A comparison is then made between the two signals. The difference signal, after being amplified by the amplifier, drives the electric heating film heating system to take corresponding actions. When the detected heating reaches the preset critical time, the electric heating film heating system starts to operate and continues to supply heat, transferring the heat to the low-temperature object—the carbon crystal–graphene heat dissipation board—through thermal conduction, thereby achieving uniform indoor temperature distribution. When the heating time reaches the preset time requirement, the difference signal becomes zero and the electric heating film heating system stops operating. It maintains a constant temperature in the room by intermittently turning on and off. The transfer function block diagram of its control system is shown in Figure 4.

The temperature control system of the graphene electric heating floor shown above is a typical closed-loop control system. It directly controls the surface temperature of the floor through a timer, thereby regulating the indoor temperature. After heating for a certain period of time and after the floor reaches a temperature suitable for human comfort, the power supply circuit is cut off to ensure safety and eliminate potential hazards.

The activation of the heating system of the electric heating film is related to a timer installed inside the floor. When the heating reaches the preset time, the electric heating film is powered off. When the floor is powered off for a period of time and the temperature drops to a critical level, the timer controls the electric heating film to resume power supply and continue heating. This cycle can maintain the surface of the floor at a comfortable temperature range, providing users with a good heating experience. The specific preset time will be discussed in subsequent research.

## 3. Heat Transfer Analysis of Graphene Electric Heating Floor

### 3.1. Heat Transfer Mechanism of Graphene-Heated Flooring

The electric heating medium of this engineered wood flooring system comprises metal carbon crystal–graphene and electric heating films. The electric heating films contain carbon fiber silver particle inkstone, which serves as the primary heating element. When plugged in, the atoms and molecules of the carbon fiber inkstone undergo intense vibrations and collisions under the influence of an electric field, thereby generating heat. The electric heating films then transfer this heat to the carbon crystal–graphene heat dissipation panel via radiation. The carbon crystal–graphene heat dissipation panel subsequently transfers the heat to the surface layer of the floor through thermal conduction. The heat generated by the electric heating films is evenly transmitted through the carbon crystal–graphene panel to the surface layer of the parquet flooring. Once the temperature of the heating core layer reaches equilibrium with the surface temperature of the floor, the decorative layer on the floor surface radiates heat at a constant temperature, transferring the heat to the indoor air through radiation [27], as follows.

(1) $$Q=Cn\left[{\left(\frac{T_{1}}{100}\right)}^{4}-{\left(\frac{T_{2}}{100}\right)}^{4}\right]F_{1}$$

In the above formula, Q is the quantity of heat, C_n_ represents the radiation coefficient and F_1_ denotes the radiation surface area of the radiator.

After gaining heat, air molecules undergo convective heat transfer with the indoor walls through their thermal motion. The heat transferred through convection is as follows:

(2) $$Q=\alpha \left(T_{w}-T_{f}\right)F$$

In the formula, Q represents the convective heat transfer, with the unit of W;

T_w_ and T_f_ represent the average temperatures of the wall and the fluid, respectively, with the unit being °C. F represents the convective heat transfer area, with the unit of m^2^. α refers to the convective heat transfer coefficient.

**Figure 5:**
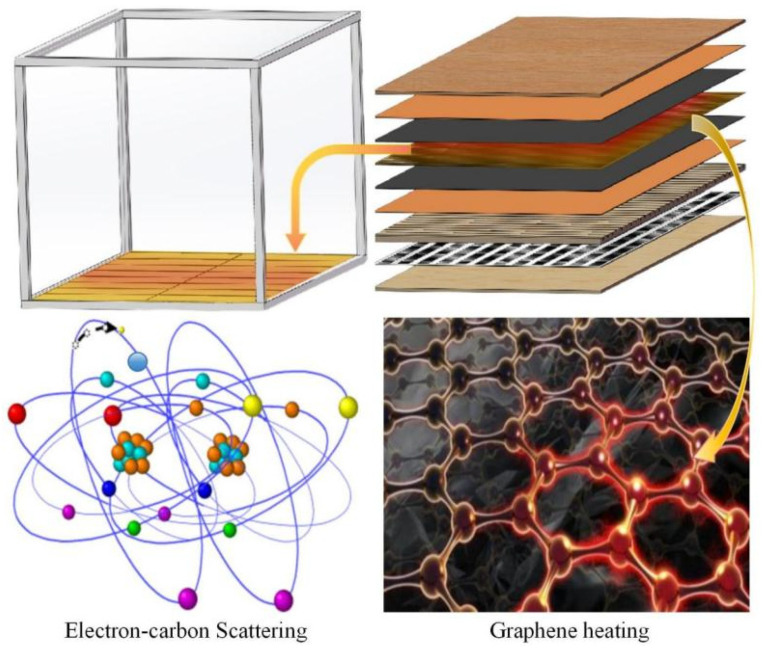
Schematic diagram of heat transfer process.

Based on the thermophysical principle that hot air is lighter and cold air is heavier, the heat radiated by the graphene electric heating floor causes the hot air in the room to continuously rise, while the cold air continuously descends to become gradually heated. By repeating this cycle, the indoor temperature continuously increases, ultimately achieving the purpose of heating the space. The heat transfer process of the aforementioned floor is shown in Figure 5.

### 3.2. Establishment of a Heat Transfer Model for Graphene-Based Heating Floor

To simplify the heat transfer process, the following assumptions are made for the model:

1. Assuming that there is good contact between layers without a temperature drop at the interlayer interface, contact thermal resistance is not introduced. Since effective heat transfer occurs only from the heating layer of the floor to the surface layer of the floor, the model is simplified as a one-dimensional heat transfer problem with heat conduction occurring only in the vertical direction, without considering other uneven heat sources and heat transfer processes;
2. Assuming that the heat transferred upward and downward by the electric heating film is equal, with each accounting for 50% of the total heat, the heat loss downward through the asbestos mesh can be neglected and the heat transferred downward is stored in the substrate layer;
3. Due to the use of low-temperature heating in the flooring, the amount of radiation generated is relatively small and can be absorbed by the solid wood floor. Therefore, the effect of thermal radiation on the surface layer of the floor is not considered;
4. Each layer of material is isotropic and homogeneous, without considering the impact of material porosity on heat transfer. The effects of water vapor, human sweat, air humidity, etc., on the heat and mass transfer process are not considered.

**Figure 6:**
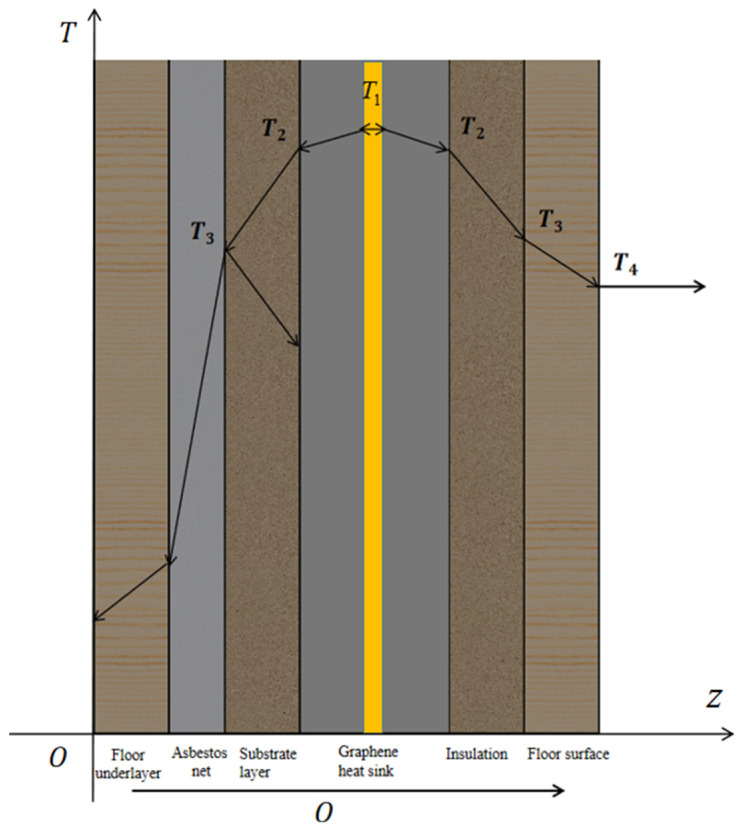
Heat transfer diagram of graphene floor.

Since heat conducts in both directions, when it reaches the lower asbestos mesh, a small portion is absorbed, while the rest is reflected back to the substrate layer and stored there. At this point, the temperature will not be conducted to the graphene heat dissipation plate but will continue to rise within the substrate layer. As the temperature of the substrate layer continues to increase, the heat emitted by the graphene plate will increasingly be conducted to the floor surface. Therefore, the amount of heat transferred along the z-axis gradually increases, and the rate of the temperature rise in the floor surface layer will gradually accelerate [28].The heat transfer modes of each layer of the floor are shown in Figure 6.

Since the internal heat source arrangement of the graphene heat dissipation board is in the form of fins for heat dissipation, the heat conduction from the core layer to both sides can be regarded as a steady-state heat transfer problem. When heat is conducted to the heat dissipation surface, it will preferentially form a uniform distribution on the surface of the heat dissipation board and then conduct to the insulation layer and the floor surface layer. Therefore, the heat transfer of the insulation layer and the floor surface layer can also be simplified as a steady-state heat transfer process. The following assumptions are made: the thickness of the intermediate heat dissipation layer is 2δ_1_; the thickness of the insulation layer is δ_2_; the thickness of the upper and lower panels is δ_3_; the total thickness of the lower solid wood floor, asbestos mesh, substrate layer, and graphene heat dissipation board is d; the total thickness of the floor is l; and the thickness of the electric heating film is negligible compared to the thickness of the floor.

Based on the heating requirements in northern winters and the concept of “warming the feet while keeping the head cool” according to theories of Chinese health preservation, the floor where people most often stay should be maintained at a temperature of 24 °C to 26 °C, with an upper limit of 28 °C. For floors where people stay for a short period of time, a temperature of 28 °C to 30 °C should be used, with an upper limit of 32 °C. For floors where no one stays, a temperature of 35 °C to 40 °C should be used, with an upper limit of 42 °C. Graphene composite heating flooring is quite common in northern households. For the surface layer of the floor where people often stay, a 32 °C electric heating film should be used as the heating temperature. For a floor surface suitable for human comfort, the upper limit is 28 °C and the temperature should be maintained at 26 °C [15]. At this time, the temperature felt by the human body is roughly between 18 °C and 22 °C, providing a refreshing warmth and comfort in the cold winter. From the perspective of energy consumption, this temperature range also saves energy and is relatively economical.

The heat source is generated by the heating of the electric heating film. Assuming that the graphene plate material is uniform, the thermal conductivity is a constant. Therefore, it can be described as a one-dimensional heat conduction equation with constant physical properties and a non-steady state, as follows:

(3) $$\frac{\partial T}{\partial t}=a\frac{\partial^{2}T}{\partial z^{2}}+\frac{\dot{\phi }}{\rho c}$$

where $a=\frac{\lambda }{\rho c}$ represents the thermal diffusivity, $T=f\left(z,t\right)$.

The initial condition is as follows: t=0, $T=T_{0}$; $t=t_{\min},T=T_{4}=22\;{}^{\circ}C$; $t=t_{\max},T=T_{4}=28\;{}^{\circ}C$; $t_{\min}\leq t\leq t_{\max}$.

The boundary condition can be regarded as the third type of boundary condition, as follows:

$${\left.\frac{\partial T(z,t)}{\partial z}\right|}_{z=l}=0$$

$$z=l,T=T_{4}$$

$$h(T_{w}-T(z,t))={\left.-\lambda \frac{\partial T(t,z)}{\partial z}\right|}_{z=l}$$

For the heat conduction problem of heterogeneous materials, it is assumed that the materials are in good contact, ignoring contact thermal resistance, and satisfying the interface continuity condition, which means that the condition of continuity of temperature and heat flux density on the interface is satisfied, as follows:

(4) $$\left\{\begin{array}{c} T(z_{i}^{-},t)=T(z_{i}^{+},t)\\ \lambda_{i}\frac{\partial T}{\partial z}(z_{i}^{-},t)=\lambda_{i+1}\frac{\partial T}{\partial z}(z_{i}^{+},t) \end{array}\right.$$

In the above formula, i represents each contact surface.

The heat transfer from the heating core layer to the floor surface layer can be regarded as a steady-state heat transfer process involving three parallel flat walls. Assuming that the thermal conductivity of the graphene heat dissipation plate, insulating layer, and solid wood floor layer are $\lambda_{1}$, $\lambda_{2}$, $\lambda_{3}$, respectively, and the four surfaces are maintained at uniform and constant temperatures $T_{1}$, $T_{2}$, $T_{3}$, $T_{4}$, the mathematical model for the heat transfer problem of the graphene heat dissipation plate can be described by the following equations:

(5) $$\frac{d^{2}T}{dz^{2}}=0$$

The boundary conditions of the first layer of the graphene heat dissipation plate are as follows:

$$\left\{\begin{array}{c} z=d+\delta_{1},T=T_{1}\\ z=d+2\delta_{1},T=T_{2} \end{array}\right.$$

The boundary conditions of the second insulating layer are as follows:

$$\left\{\begin{array}{c} z=d+2\delta_{1},T=T_{2}\\ z=d+2\delta_{1}+\delta_{2},T=T_{3} \end{array}\right.$$

The boundary conditions of the third solid wood veneer layer are as follows:

$$\left\{\begin{array}{c} z=d+2\delta_{1}+\delta_{2},T=T_{3}\\ z=d+2\delta_{1}+\delta_{2}+\delta_{3},T=T_{4} \end{array}\right.$$

The differential equation above is integrated twice to obtain the following general solution:

$$T=c_{1}z+c_{2}$$

By substituting the first-layer boundary condition into the general solution, we obtain the temperature distribution as follows:

$$T=\frac{T_{2}-T_{1}}{\delta_{1}}z+T_{1}-\frac{T_{2}-T_{1}}{\delta_{1}}(d+\delta_{1})$$

Since the boundary temperature and thickness are constant, the temperature distribution is linear. Therefore, the slope of the temperature distribution curve is as follows:

$$\frac{dT}{dz}=\frac{T_{2}-T_{1}}{\delta_{1}}$$

By substituting this into Fourier’s law, $q=-\lambda \frac{dT}{dz}$, we can obtain the relationship between the heat flux density and heat quantity, as follows:

(6) $$q=\frac{\lambda (T_{1}-T_{2})}{\delta_{1}}\;Q=A\frac{\lambda_{1}(T_{1}-T_{2})}{\delta_{1}}$$

The thermal resistance of the first layer of graphene heat dissipation plate is as follows:

(7) $$R_{1}=A\frac{T_{2}-T_{1}}{Q}=\frac{T_{2}-T_{1}}{q}$$

Since the three flat walls are closely fitted together, their total thermal resistance should be equal to the sum of the thermal resistances of each individual part, as follows:

$$R_{sum}=R_{1}+R_{2}+R_{3}$$

among $\left\{\begin{array}{c} R_{1}=\frac{T_{1}-T_{2}}{q}=\frac{\delta_{1}}{\lambda_{1}}\\ R_{2}=\frac{T_{2}-T_{3}}{q}=\frac{\delta_{2}}{\lambda_{2}}\\ R_{3}=\frac{T_{4}-T_{3}}{q}=\frac{\delta_{3}}{\lambda_{3}} \end{array}\right.$, $R_{sum}=\frac{T_{1}-T_{4}}{q}$ Substituting the above expression we can obtain the total heat flux density, as follows:

(8) $$q=\frac{\Delta T}{R_{sum}}=\frac{T_{1}-T_{4}}{\frac{\delta_{1}}{\lambda_{1}}+\frac{\delta_{2}}{\lambda_{2}}+\frac{\delta_{3}}{\lambda_{3}}}$$

For the heat transfer process of solid wood composite graphene flooring a model is established as follows:

$$\left\{\begin{array}{l} \mathrm{Governing}\;\mathrm{equation}:\frac{\partial T}{\partial t}=a\frac{\partial^{2}T}{\partial z^{2}}+\frac{\dot{\phi }}{\rho c}\\ \mathrm{Heat}\;\mathrm{transfer}\;\mathrm{equation}:\frac{d^{2}T}{dz^{2}}=0\\ \mathrm{Boundary}\;\mathrm{condition}:\left\{\begin{array}{l} t=0,T=T_{0};t_{\min}\leq t\leq t_{\max};\\ t=t_{\min},T=T_{4}=22\;{}^{\circ}C;t=t_{\max},T=T_{4}=28\;{}^{\circ}C\\ \frac{\partial T(z,t)}{\partial z{|}_{z=l}}=0\\ h(T_{w}-T(z,t))=-\lambda \frac{\partial T(t,z)}{\partial z}{|}_{z=l} \end{array}\right.\\ \mathrm{Contact}\;\mathrm{surface}\;\mathrm{equation}:\left\{\begin{array}{c} T(z_{i}^{-},t)=T(z_{i}^{+},t)\\ \lambda_{i}\frac{\partial T}{\partial z}(z_{i}^{-},t)=\lambda_{i+1}\frac{\partial T}{\partial z}(z_{i}^{+},t) \end{array}\right. \end{array}\right.$$

### 3.3. Solving the Model

The total thickness of the parquet floor is 18 mm. The electric heating film is 0.5 mm thick. The heat dissipation graphene layer ranges from 2 to 6 mm in thickness, with an insulation layer of 0.5 mm, a solid wood floor base material layer spanning from 7~9 mm, and an asbestos insulation layer of 2 mm. Under the condition of ensuring a minimum temperature of 22 °C and a maximum temperature of 28 °C for the floor layer, the thicknesses of the solid wood floor surface layer and the graphene layer are adjusted using the finite difference method [29] to obtain the optimal thickness for the floor surface layer. The heat transfer model is discretized using an explicit difference scheme, and the temperature at the (n + 1)th time step relies on the temperature information from the previous layer. In the discretization scheme of the control equation, there is only one unknown quantity $T_{i}^{n+1}$, as follows:

$$\left\{\begin{array}{l} \mathrm{Governing}\;\mathrm{equation}:\frac{T_{i+1}^{n}-T_{i}^{n}}{\Delta t}=a_{j}\frac{T_{i+1}^{n}-2T_{i}^{n}+T_{i-1}^{n}}{\Delta z_{j}}+\frac{\phi_{i+1}^{n}-\phi_{i}^{n}}{\rho_{j}c_{j}\Delta z_{j}\Delta t}\\ \mathrm{Boundary}\;\mathrm{condition}:\left\{\begin{array}{l} \frac{T_{i}^{n+1}-T_{i}^{n}}{\Delta z_{j}}=0\\ h_{j}(T_{end}^{4}-T_{o}^{4})=-\lambda_{1}\frac{T_{i}^{n+1}-T_{i}^{n}}{\Delta z_{j}} \end{array}\right.\\ \mathrm{Contact}\;\mathrm{point}:\frac{1}{2}(\Delta z_{j}\rho_{j}c_{j}+\Delta z_{j+1}\rho_{j+1}c_{j+1})\frac{T_{i}^{n+1}-T_{i}^{n}}{\Delta t}=\lambda_{j}\frac{T_{i-1}^{n}-T_{i}^{n}}{\Delta z_{j}}+\lambda_{j+1}\frac{T_{i+1}^{n}-T_{i}^{n}}{\Delta z_{j+1}} \end{array}\right.$$

For explicit difference schemes, the discrete solution of unsteady heat transfer processes requires consideration of the stability conditions for the solution. The aforementioned explicit difference scheme indicates that the temperature at time step n + 1 at spatial node *i* is influenced by the adjacent points on both sides, and stability constraints (Fourier grid number limit) must be met, so that otherwise unreasonable oscillatory solutions may occur, as follows:

$$\left\{\begin{array}{c} F_{O_{\Delta }}=\frac{\lambda \Delta t}{\rho c{\left(\Delta z\right)}^{2}}\\ F_{O_{\Delta }}\leq \frac{1}{2} \end{array}\right.$$

**Figure 7:**
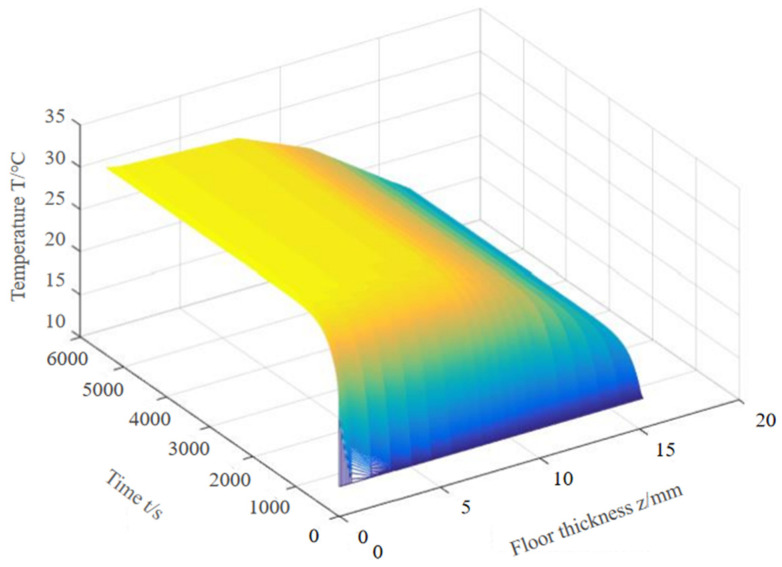
Temperature change under different thickness and time.

After the time–space discretization of the unsteady heat transfer model, it can be solved layer by layer at time nodes and spatial nodes based on the boundary conditions and initial conditions. With the help of MATLAB 2015Ra software, the temperature distribution at different thicknesses was obtained and is shown in Figure 7.

**Figure 8:**
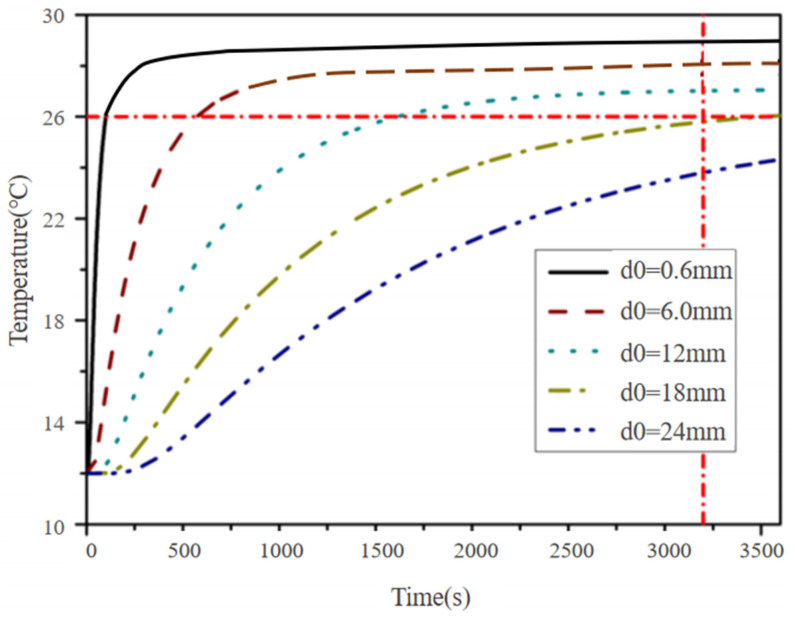
Temperature changes on the floor surface under different thicknesses.

Five different thicknesses, namely, 0.6 mm, 6 mm, 12 mm, 18 mm, and 24 mm, were selected. The thermal conductivity was the average value of the thermal conductivity of each layer. The relationship between the temperature rise on the surface of the floor and time is shown in Figure 8.

**Figure 9:**
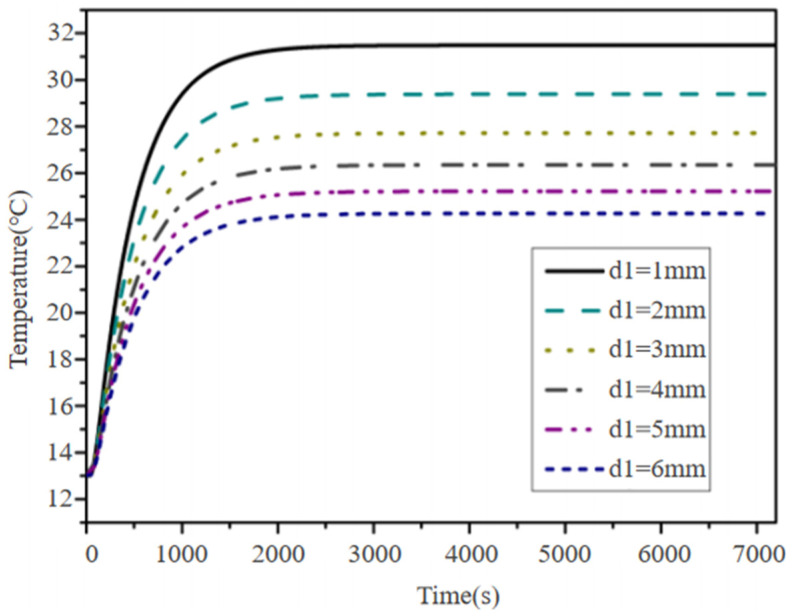
Temperature variations under different floor surface thicknesses.

The temperatures of the insulating layer and the graphene heat dissipation layer was fixed by adjusting the thickness of the floor surface layer. The relationship curve between the temperature rise in the floor surface layer and time under a heating temperature of 32 °C is shown in Figure 9.

**Figure 10:**
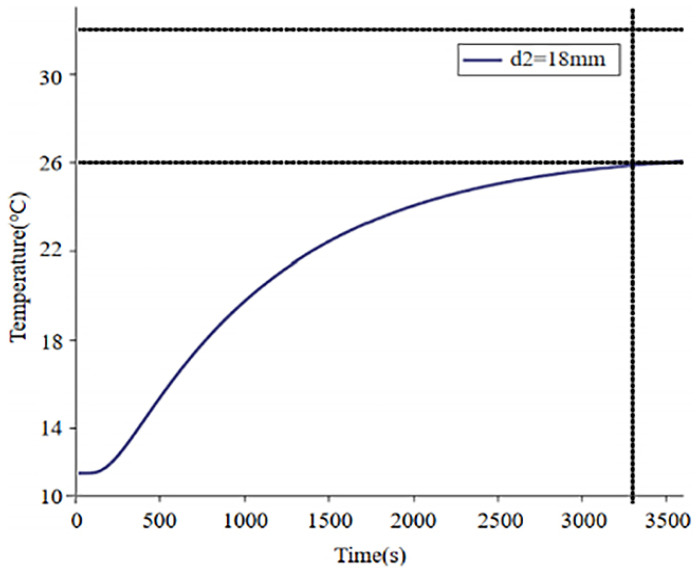
Time–temperature variations in 18 mm-thick floor.

Based on the requirements of indoor temperature changes, the optimal thickness of the floor surface layer was selected as 4 mm. The relationship between the surface temperature of the floor and time was obtained shown in Figure 10.

Based on the aforementioned results, the thickness of each layer in the graphene–electric heated solid wood composite floor is as follows: a surface layer of 4 mm; a graphene layer of 4 mm; a substrate layer of 8 mm; and a bottom layer of 2 mm. The asbestos mesh insulation layer is embedded beneath the substrate, without contributing to the total thickness, resulting in a total floor thickness of 18 mm.

**Figure 11:**
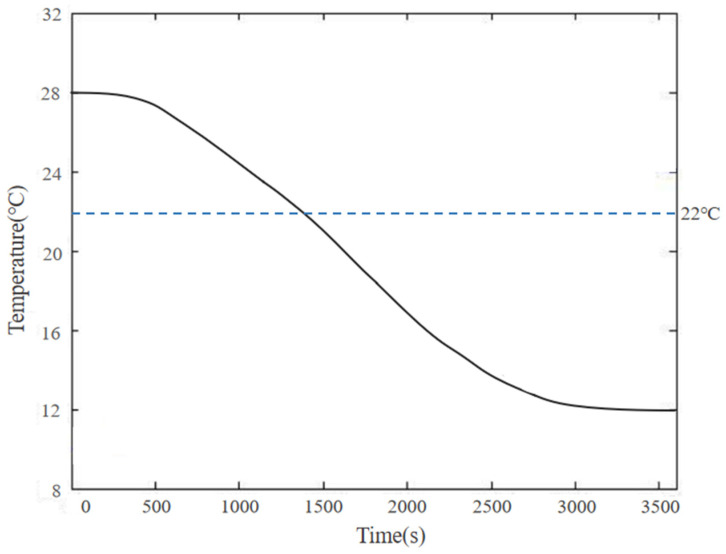
Floor cooling temperature variation curve.

When the temperature of the floor reaches 28 °C, the control system cuts off power to the electric heating film. The heat stored in the floor substrate layer will be continuously released into the indoor air through convective heat transfer, causing the temperature of the floor surface to gradually decrease. With a fixed floor thickness and convective heat transfer coefficient, the temperature change in the floor surface reaching 28 °C for power-off cooling is shown in Figure 11.

After fitting, it was found that for an 18 mm-thick floor, the time required to heat it to 22 °C is 1637 s; the time required to heat to 26 °C is 3342 s; and the time required to heat to 28 °C is 6533 s. For the convenience of setting a timer, the heating time was rounded up to minutes, resulting in a time of 27 min (1620 s) to reach 22 °C; 56 min (3360 s) to reach 26 °C; and 109 min (6540 s) to reach 28 °C. Therefore, after the floor is activated, it can be powered off after heating for 109 min. After the floor is powered off, the temperature drops to 22 °C after 1486 s, totaling 25 min (1500 s). After the floor is powered off for 25 min, the control system heats it again for 81 min. This cyclic system ensures that the surface temperature of the floor remains between 22 °C and 28 °C, providing users with the most comfortable experience.

## 4. Temperature Comparison Experiment

To verify the heat transfer efficiency and energy storage effect of graphene-based electric heating flooring, a temperature comparison experiment was designed, with ordinary electric heating flooring and the solid wood composite graphene-based electric heating flooring as the experimental subjects. By comparing the differences in heat transfer between the two after being powered on, conclusions were drawn. The experiment was conducted in November in Harbin, Heilongjiang Province, China, located between 44°04′ and 46°40′ N latitude with an outdoor temperature of −10 °C. During the heating season, to avoid excessive heating interference with the experimental results, an experimental site with poor heating effects was selected. The experimental site was chosen at the laboratory on the first floor of the Forestry and Woodworking Machinery Engineering Technology Center of Northeast Forestry University in Harbin. The laboratory was unmanned for most of the winter, with an initial indoor temperature of 14 °C. The indoor–outdoor heat transfer coefficient was taken as 10 W/(m^2^·K); the electric heating film power was 400 W; and a composite PCM was selected as the phase change material [30]. Its physical properties are shown in Table 2.

**Figure 12:**
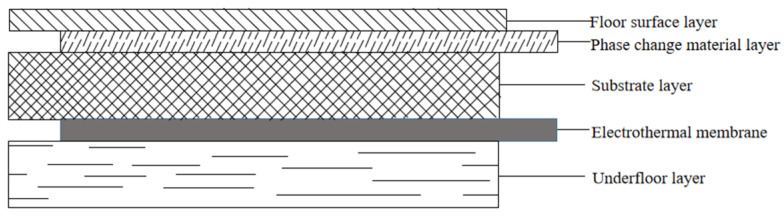
Floor structure drawing.

The comparison object was a PCM electric heating floor with a thickness of 18 mm. The surface layer was made of eucalyptus veneer with a thickness of 4 mm, and the substrate layer consisted of an 8 mm-thick sawn pine veneer. The electric heating film was heated at a low temperature and the bottom plate was made of pine with a thickness of 2 mm. The basic structure is shown in Figure 12.

**Figure 13:**
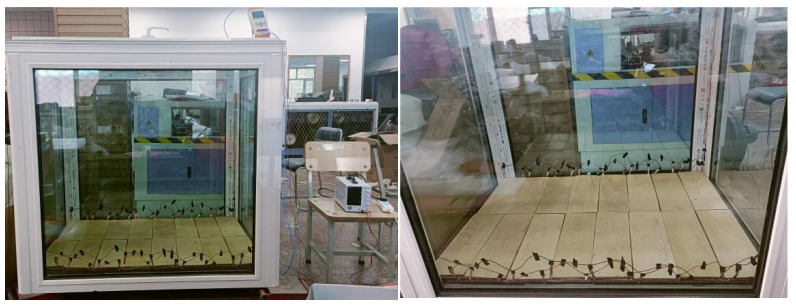
Test scenario of graphene electric heating floor.

Referring to the relevant standards in ISO 12567-1-2010 [31], we employed the “hot box method” to test the temperature characteristics of the flooring. The team constructed a thermal insulation box, shown in Figure 13, with an initial internal temperature identical to that of the room temperature. The dimensions of the thermal insulation box were 1 m × 1 m × 1.5 m. A thermocouple temperature gauge was employed to monitor temperature variations on the surface and bottom layers of the floor over a unit time period. The selected indoor temperature was maintained at a constant 14 °C ± 2 °C after heating, with a humidity level of 20 ± 5%. Additionally, a humidifier was placed indoors to ensure minimal humidity fluctuations post heating. Three ordinary electric heating floors and three solid wood composite graphene electric heating radiant panels were assembled into two groups, with each group consisting of three pieces. These two groups were laid flat on opposite sides of the laboratory, facing upwards. Six temperature acquisition points were evenly distributed on the floors, while three temperature acquisition points were arranged vertically indoors, spaced 0.2 m apart. After being powered on for 1000 s, the surface temperature of the graphene electric heating floor gradually increased as it was heated, leading to a growing temperature difference between the inside and outside. After stopping the heating process for 0.5 h, the surface temperature of the floor naturally cooled down.

**Figure 14:**
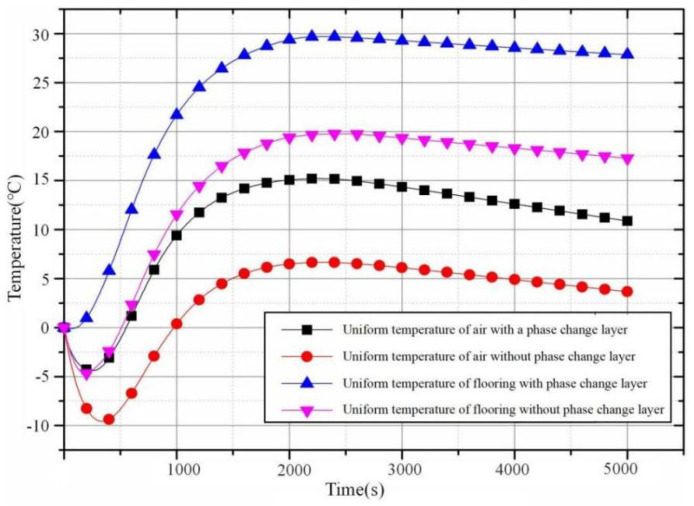
Air and floor temperature changes.

The temperature–time curves in Figure 14 show the temperature of the floor surface and the indoor environment in the curve graph.

As can be seen from the graph, the temperature of the floor with the phase-change layer is higher than that without a phase-change layer, which is beneficial to indoor heating. It can be observed from the graph that there is a temperature drop at the initial stage after power-on, which is due to the heating speed at the initial stage being lower than that of the cooling speed of the outdoor low temperature. After 1000 s, the heating stops, and both the floor and air temperatures continue to rise, which is due to the heat released by the system’s own heating and the energy storage of the composite floor. When the residual heat of the system dissipates and the temperature reaches its highest point, the temperature begins to slowly decrease, and the temperature of the floor surface remains above 20 °C for a considerable period of time. This indicates that the energy storage effect of the energy storage floor is good, and that it can also maintain a comfortable indoor temperature after the power is switched off.

**Figure 15:**
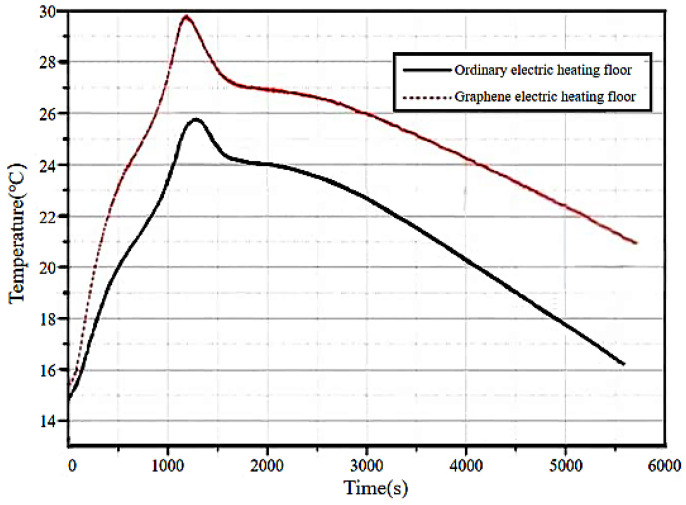
Comparison of different floor temperature time changes.

The surface temperature changes in the ordinary electric heating flooring and composite graphene electric heating flooring after being powered on for 1000 s are shown in Figure 15.

As can be seen in Figure 15, the flooring will continue to heat up for a period of time after the heating is stopped. The energy storage effect of graphene electric heating flooring is better. Within 1 h after experiencing the peak, the ordinary flooring quickly cools down to below 20 °C, while the composite graphene electric heating flooring cools down relatively slowly and the surface temperature of the flooring remains above 20 °C within 1 h. Therefore, it can be seen that the graphene–electric heating solid wood composite flooring has an excellent energy storage effect. It can save more energy while providing a comfortable living environment.

Through comparative experiments, it was verified that the graphene-based electric heating solid wood composite flooring exhibits superior heat transfer efficiency and energy storage capabilities. This indicates that this approach possesses considerable scientific value and market potential. Additionally, it offers a safer, cleaner, more comfortable, and healthier heating method for cold northern winters.

## 5. Conclusions

(1) This study has designed a new type of graphene-heated composite flooring which incorporates an asbestos mesh insulation layer into the traditional sandwich structure, effectively reducing heat loss and ensuring that heat is conducted into the room as much as possible. At the same time, a temperature control system was designed for this flooring, enabling autonomous temperature regulation. The experimental design showed that this flooring has good heat storage effects and can continue to release heat for a certain period of time after the power is turned off;
(2) This study established a three-dimensional model of a graphene electric heating floor and simulated the heat transfer process using the finite difference method. We found that for a thickness of 18 mm, it takes approximately 27 min (1620 s) for the surface to reach 22 °C; 56 min (3360 s) to reach 26 °C; and 109 min (6540 s) to reach 28 °C. After turning off the power, it takes 25 min (1500 s) for the floor surface to return to 22 °C;
(3) For this type of graphene electric heating floor, experiments were designed to show that the addition of phase change materials to the composite graphene electric heating floor resulted in improved heating effects. Therefore, incorporating phase change materials during the preparation of the floor will enable it to possess excellent heat conduction and storage properties.

## Figures and Tables

**Table 1:** Comparison of thermal conductivity of various materials.

Material	Graphene	Ag	Cu	Al	Steel
Thermal conductivity	5000 W/(m·K)	429 W/(m·K)	401 W/(m·K)	226 W/(m·K)	73.3 W/(m·K)

**Table 2:** Physical parameters of phase change materials.

Phase Transition Temperature/(K)	Latent Heat/(kJ·kg^−1^)	Specific Heat Capacity/kJ·(kg·K)^−1^	Density/kg·m^−3^	Thermal Conductivity/W (m^2^·K)
302	234.33	3.71	1034	2.58

## Data Availability

The data presented in this study are available on request from the corresponding author due to privacy.
